# Risk factors for new-onset diabetes mellitus after kidney transplantation (NODAT): a Brazilian single center study

**DOI:** 10.20945/2359-3997000000084

**Published:** 2018-10-01

**Authors:** Camila Lima, Amanda Grden, Thelma Skare, Paulo Jaworski, Renato Nisihara

**Affiliations:** 1 Universidade Evangélica Departamento de Medicina Curitiba PR Brasil Universidade Evangélica, Departamento de Medicina, Curitiba, PR, Brasil; 2 Universidade Positivo Universidade Positivo Departamento de Medicina Curitiba PR Brasil Universidade Positivo, Departamento de Medicina, Curitiba, PR, Brasil

**Keywords:** New-onset diabetes after transplantation (NODAT), diabetes mellitus, renal transplant, calcineurin inhibitors

## Abstract

**Objectives:**

This study aims to verify the new-onset diabetes after kidney transplant (NODAT) incidence in recipients within 1 year after kidney transplantation from a single center in Southern Brazil and to assess the associated conditions.

**Subjects and methods:**

A retrospective study of 258 post-renal transplant patients was performed. Demographic (gender, age, ethnic background) and clinical (origin of graft, associated infections, body mass index (BMI) at transplant time and 6 and 12 months after, causes of renal failure, and comorbidities) data were analyzed. All patients were on tacrolimus, mycophenolate mofetil, and prednisone treatment. Patients with and without NODAT were compared.

**Results:**

A NODAT incidence of 31.2% was noted 1 year post transplantation. In the univariate analysis, patients with NODAT were older (p = 0.001), mostly had African–American ethnic background (p = 0.02), and had renal failure secondary to high blood pressure (HBP) (p = 0.001). The group of patients with NODAT also had more incidence of post-transplant HBP (p = 0.01), heart failure (p = 0.02), and dyslipidemia (p = 0.001). Logistic regression showed that African–American ethnic background, post-transplant HBP, and dyslipidemia were independently associated with NODAT.

**Conclusion:**

This study shows a NODAT incidence that is greater in patients with African–American ethnic background and that is associated with HBP and dyslipidemia.

## INTRODUCTION

New-onset diabetes after transplant, or NODAT, is one of the major complications after solid organ transplantation (1,2). There is a five- to six-fold higher incidence of new-onset diabetes mellitus (DM) in the first year post kidney transplantation in comparison to waiting-listed patients (3). This complication is considered to lead to graft failure and to promote cardiovascular disease, one of the main causes of death in transplant recipient (4). A large meta-analysis has shown that NODAT is present in 2%–50% of patients, 1 year after the transplant procedure (5).

The pathophysiology of NODAT closely mimics type 2 DM: insulin resistance and insulin hyposecretion develop in both situations. Nevertheless, in NODAT, insulin hyposecretion seems to be a crucial determining factor in glucose tolerance deterioration (4).

Several risk factors were known to be associated with NODAT, such as ethnic background, family history of diabetes, sedentary lifestyle, high body mass index (BMI), and cytomegalovirus and hepatitis C virus (HCV) infection. A high dose of glucocorticoids and the use of immunosuppressive regimen with calcineurin inhibitors may also be associated with this condition (6-8). Numakura and cols. (9) found that genetic factors such as certain vitamin D receptor haplotypes lead to increased risk of NODAT. Kim and cols. (10) found that Toll-like receptor (TLR)-4 and TLR-6 gene polymorphisms were significantly associated with NODAT, showing that the innate immune system and inflammation via TLR activation may have an important role in the pathogenesis of this complication. The results of some studies indicate that several genes, such as interleukins, transcription factor 7-like 2, solute carrier family 30 (zinc transporter), member 8 genes (SLC30A8), matrix metalloproteinases (MMPs), and chemokine (C–C motif) ligand 5 (CCL5) genes, were related with the development of NODAT (11-15).

Genetic background may influence the prevalence of NODAT in different populations (6). The increased survival of renal transplant patients requires adequate attention as its complication may interfere with patients’ survival and quality of life (16,17). Aiming to know the prevalence of NODAT and its risk factors in the region, a cohort of kidney transplant recipients from Southern Brazil were studied.

## SUBJECTS AND METHODS

This retrospective study was approved by the local Committee of Ethics in Research (56934016.4.0000.0103). Charts of kidney transplant patients from a single center, a university evangelical hospital, located in the city of Curitiba, Paraná, Brazil, registered from July to December 2015, were reviewed for epidemiological, clinical, and treatment profile.

Patients under 18 years of age, those with DM prior to transplantation, those who had first-degree relatives with DM, and those who had used immunobiological drugs or had graft rejection were excluded.

The study sample included 258 patients (141/268 or 52.6% males, with median age of 43 years; range 18–75 years). All patients were on standard treatment, with prednisone, mycophenolate mofetil, and tacrolimus, which is determined by the Brazilian Health Ministry, Ordinance 1.168, in 2004.

The following data of all patients were reviewed retrospectively for 12 months: BMI prior to transplantation and six and twelve months post transplantation, causes of renal failure prior to transplantation, graft origin (either from deceased or living donor), and occurrence of hepatitis C and CMV infections. The diagnosis of DM was made according to the American Diabetes Association, through the analysis of three altered values of blood glucose, which was done by the transplant doctor and was registered in the patient medical record 12 months after transplantation (18).

The obtained data was collected in frequency and contingency tables. The epidemiological and clinical profiles between patients with and without NODAT were compared using the Fisher exact test and chi-square tests for nominal data and unpaired t-test and Mann–Whitney test for numeric data. To assess variables independently, logistic regression was used. The significance level adopted was 5%.

## RESULTS

The description of clinical, epidemiological, and treatment data of kidney transplant patients being studied are shown in Table 1.

**Table 1:** Clinical, epidemiological and treatment data of kidney transplanted patients (n = 258)

Variable		n	(%) or central tendency
Ethnic background (auto declared)	Caucasians	209	35;78.6
	Asian	1	0.3
	Afro descendants	53	20.9
Causes of renal failure	Chronic glomerulonephritis	125	51.9
	Hypertension	43	16.6
	Polycystic kidney disease	21	8.1
	Pyelonephritis and litiasis	12	4.6
	IgA nephropathy	11	4.2
	Others	37	14.6
	6 months post transplantation		24.2 (22.2-28.5)
	12 months post transplantation		26.1 (23.1-29.7)
Graft type	Cadaveric	121	46.8
	Living donor (relative)	98	37.9
	Living donor (not relative)	37	14.3
	Unknown	2	0.7
Co-morbidities	Arterial hypertension	229	87.5
	Dyslipidemia	38	14.3
	Ischemic heart disease	10	3.8
	Heart failure	3	1.1

BMI: body mass index; CMV: cytomegalovirus.

Only three patients had HCV infection. In this sample, the NODAT incidence was 31.2%.

Table 2 shows the results obtained between patients with and without NODAT were compared. It is noticeable that elder patients, African–American background patients, and the group with hypertension and dyslipidemia had a higher NODAT incidence. Patients with NODAT also experience more hypertension and less chronic glomerulonephritis, which are causes of renal failure that required transplantation.

**Table 2:** Comparison of renal transplanted patients according to presence or not of NODAT (New Onset Diabetes After Transplantation)

	With NODAT 31.2%	Without NODAT 68.8%	P
Median age (years)	48 (37-56)	41 (26-51)	0.001
Ethnic background	Afro descend - 30.2% Caucasian - 69.7%	Afro descend - 17.5% Caucasian - 82.4%	0.02*
Male gender	62%	52.8%	0.17
Causes of renal failure	Glomerulonephritis - 35.8% Hypertension - 28.2% Others - 35.8%	Glomerulonephritis - 56.6% Hypertension - 12.0% Others - 31.3%	0.001**
Graft donor	Deceased - 53.1% Living related - 36.7% Living not related - 10.1%	Decesead - 44.7% Living related - 15.6% Living not related - 39.2%	0.34
Median BMI (kg/m^2^) pre transplant	24.9 (22.0-27.9)	24.5 (21.5-28.1)	0.49
Mean BMI (kg/m^2^) 6 months post-transplant	25.5 ± 4.1	25.4 ± 4.2	0.78
Mean BMI (kg/m^2^) 12 months post-transplant	26.4 ± 4.7	26.3 ± 4.5	0.89
Associated arterial hypertension	97.4%	85.6%	0.01#
Associated dyslipidemia	25.3%	9.7%	0.001##
Associated heart failure	3.7%	0	0.02§
Associated coronary insufficiency	5.06%	9.7%	0.50
CMV infection	1.2%	4.02%	0.44

BMI: body mass index; CMV: cytomegalovirus.

* Odds ratio or OR: 2.04; 95% confidence interval (CI): 1.08-3.8;

** HAS versus glomerulonephritis with OR: 3.6; 95% CI: 1.7-7.7.

# OR: 4.3; 95% CI: 1.2-14.7.

## OR: 3.1 (1.4-6.3).

§ OR: 15.9; 95% CI: 0.8-313.1.

When all variables with p < 0.1 were studied through logistic regression, it was found that African–American ethnic background (p = 0.03; OR = 2.08; 95% CI = 1.05-4.14), dyslipidemia (p = 0.03; OR = 2.08; 95% CI = 1.09–5.12), and hypertension (p = 0.04; OR = 4.7; 95% CI = 1.04–21.5) were independently associated with NODAT.

## DISCUSSION

In the present study, which included 258 renal post transplanted recipients, all in the same therapeutic regimen, there was a NODAT incidence of 31.2% in the first year after the transplant procedure. It is noteworthy that individuals with DM in the first-degree relatives in the sample were excluded, so the possible occurrence of a common type 2 DM was minimized. In addition, it was observed that NODAT was independently associated with factors such as African–American background, arterial hypertension, and dyslipidemia.

Other authors have found that a myriad of factors affect the development of NODAT, such as high BMI, the use of calcineurin inhibitors and corticosteroids, old age, and cytomegalovirus (CMV) and hepatitis C infection (1). BMI alone was not linked to the appearance of this complication in the sample. However, the sample's mean BMI ranged from 24 to 26 kg/m^2^ which is close to the accepted normal range. Therefore, obesity, which is widely linked to DM, was underrepresented in the sample. This may explain the diversity of the results found. Additionally, CMV infection could not be associated with NODAT in this study, considering the very low incidence of infection in patients. The same occurs with HCV infection. However, literature concerning HCV infection and NODAT is ambiguous, with some authors supporting higher prevalence of these findings (19) and others denying it (20,21). In this sample, two of three patients with HCV infection developed DM.

NODAT's association with African–American background was also described by other authors (19). This association was also observed in the Brazilian sample, regardless of the fact that Brazilian people have a highly mixed ethnic background.

Tacrolimus is considered one of the main NODAT causes, (22) and all of the patients were receiving it. A calcineurin inhibitor has been considered the cornerstone of immunosuppressive regimens in renal transplant (22). It inhibits T-cell activation, thereby inhibiting interleukin-2 syntheses, and leads to a failure of T-cell clonal expansion (23). Several authors have shown that patients treated with tacrolimus have higher NODAT incidence when compared to those who were treated with cyclosporine (1,24). Nonetheless, tacrolimus was shown to be superior to cyclosporine in terms of patient mortality, graft loss, and hypertension development (24).

The mechanism involved in tacrolimus-induced NODAT includes diminished insulin secretion, as calcineurin is expressed in pancreas β cells. This medication disrupts mitochondrial permeability, causes inhibition of NFAT transcription factor, and targets cAMP-responsive element-binding protein transcriptional coactivator (3). In addition, it inhibits glucose uptake from muscular cells and adipocytes (3). Hypomagnesemia is one of the proposed mechanisms for calcineurin-associated glucose intolerance, due to magnesium which is essential for glucose transport and pancreatic insulin secretion, and is involved in the post-receptor insulin signaling (25).

NODAT is associated with lower survival of transplanted recipients, and it is an independent risk factor linked to graft failure (22). Furthermore, classic DM complications are seen in patients with NODAT such as peripheral neuropathy, ketoacidosis, hyperosmolar coma, and even biopsy-proven diabetic nephropathy (22,26). Serum creatinine levels are significantly higher in patients with NODAT compared to those without it after 5 years of transplantation (22,26). All these complications lead to increased mortality, mainly due to cardiovascular events (22,26). Cosio and cols. (27) demonstrated that the 5-year cumulative incidence of cardiovascular events was correlated with fasting glucose levels seen 1 year after transplantation. It was found that a higher rate of hypertension and dyslipidemia in the NODAT sample is known to be a risk factor for atherosclerotic disease and cardiovascular events.

This is a retrospective study, which is also considered one of the limitations. It is also possible that the occurrence of NODAT would be higher in longer periods of observation.

In conclusion, this study shows a higher NODAT incidence in kidney transplant recipients with African–American ethnic background. Moreover, NODAT is associated with higher blood pressure levels and dyslipidemia in all transplant recipients, regardless of the ethnic background. Prospective and multicentric studies may be useful to better understand and prevent NODAT.
